# An unexpected embolic source: mitral annular calcification presenting with stroke and myocardial infarction—case report

**DOI:** 10.1093/ehjcr/ytag793

**Published:** 2026-10-23

**Authors:** Jessica V Yao, Pavithra Jayadeva, Michael O’Keefe, Kai En Leong, Matthew J Brooks

**Affiliations:** Department of Cardiology, The Royal Melbourne Hospital, 300 Grattan Street, Parkville, Victoria 3050, Australia; Department of Medicine, Dentistry and Health Sciences, The University of Melbourne, Grattan Street, Parkville, Victoria 3010, Australia; Department of Cardiology, The Royal Melbourne Hospital, 300 Grattan Street, Parkville, Victoria 3050, Australia; Department of Cardiothoracic Surgery, The Royal Melbourne Hospital, 300 Grattan Street, Parkville, Victoria 3050, Australia; Department of Cardiology, The Royal Melbourne Hospital, 300 Grattan Street, Parkville, Victoria 3050, Australia; Department of Cardiology, The Royal Melbourne Hospital, 300 Grattan Street, Parkville, Victoria 3050, Australia

**Keywords:** Echocardiography, Imaging modalities, Cardiac computed tomography, Cardiac surgery, Mitral annular calcification

Learning points

- Caseous mitral annular calcification (MAC) is a rare and typically benign condition but may occasionally lead to life-threatening embolic complications.
- Multimodal imaging is essential for distinguishing caseous MAC from other differential diagnoses, including tumours and infective vegetations.

## Introduction

Caseous mitral annular calcification (MAC) is a rare variant of MAC. Most patients with caseous MAC are asymptomatic. Embolic complications are uncommon. Multimodal imaging plays a key role in both the diagnosis and management of caseous MAC.

## Summary figure

**Figure:**
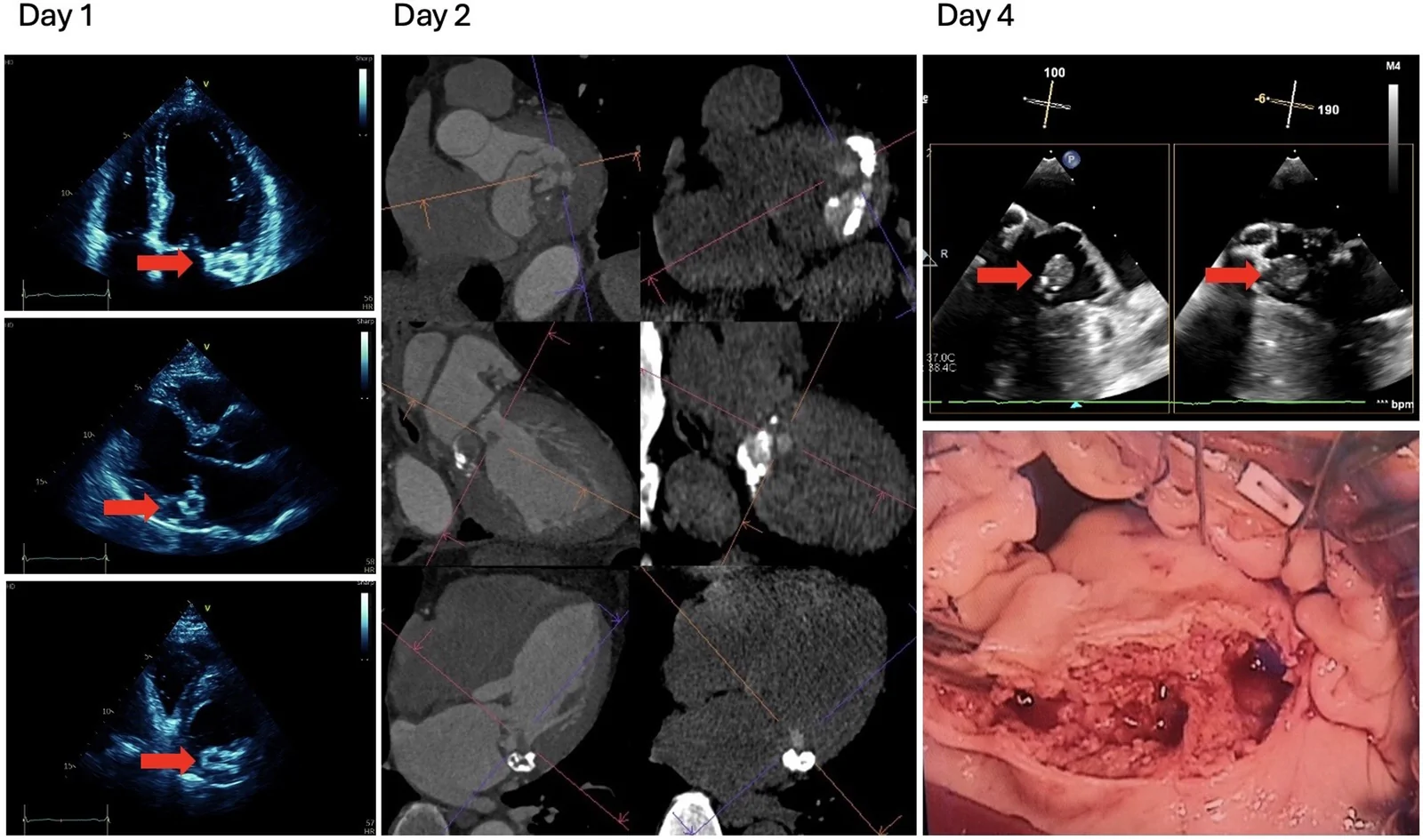


## History of presentation

A 78-year-old male presented with a left eye scotoma, right facial droop, and dull central chest pain. Past medical history included hypertension, dyslipidaemia, mild aortic regurgitation, and mitral regurgitation. Troponin peaked at 1013 (<20 g/L). Inflammatory markers were normal. Electrocardiogram demonstrated sinus rhythm with no ischaemic changes. Computed tomography (CT) of the brain was unremarkable. Magnetic resonance imaging of the brain demonstrated bilateral, multi-territory acute punctate infarcts in the cerebellum, pons, and basal ganglia, concerning for embolic infarcts. No vessel occlusion nor significant stenosis was identified in the circle of Willis. Transthoracic echocardiogram demonstrated mild segmental systolic dysfunction with an ejection fraction of 49% and hypokinetic basal inferolateral and inferior walls. There was moderate mitral regurgitation and a bright round echogenic structure with an echolucent core noted on the posterior mitral valve leaflet and annulus, thought to represent MAC (*Figure 1*). Aspirin and therapeutic anticoagulation were initiated for the management of the embolic strokes and myocardial infarction (MI). Subsequent coronary angiogram demonstrated minor coronary artery disease. Cardiac CT confirmed severe caseous MAC at the lateral annulus (*Figure 2*). Pre-contrast (right panels) and post-contrast (left panels) images demonstrated a high-attenuation globular excrescence extruding from the P1 annulus (crosshairs) into the left ventricle. Otherwise, no thrombus was seen in the left ventricle or left atrial appendage (LAA). Telemetry demonstrated no atrial fibrillation or flutter. The stroke and non-ST-segment elevation myocardial infarction (NSTEMI) were attributed to embolization from ruptured caseous MAC, and anticoagulation was ceased. Alternative causes of myocardial injury, including Takotsubo cardiomyopathy and an acute coronary syndrome, were considered. However, the regional wall motion abnormalities corresponded to a discrete coronary artery territory, making Takotsubo cardiomyopathy less likely. Furthermore, angiography demonstrated no evidence of plaque rupture or significant coronary stenosis, reducing the likelihood of an atherosclerotic event. Thus, the MI was presumed to be secondary to coronary embolization. Transoesophageal echocardiogram (TOE) demonstrated no significant aortic atheroma or structural abnormalities, including fibroelastoma, patent foramen ovale, or intracardiac masses. Carotid imaging and extended ambulatory rhythm monitoring were not performed. Thus, significant carotid disease and paroxysmal atrial fibrillation could not be definitively excluded. He was taken for surgery (*Figure 3*) with intraoperative TOE guidance, which confirmed MAC with a globular excrescence at the P1 annulus (*Figure 4*). He underwent successful decalcification of MAC using a Cavitron Ultrasonic Surgical Aspirator, a bioprosthetic mitral valve replacement, reconstruction of the atrioventricular groove, and LAA excision. Post-operative course was uncomplicated. Given the presence of a bioprosthetic valve, significant cardiovascular risk factors, and evidence of minor coronary atherosclerosis, long-term aspirin therapy was continued. He remains well 3 months post-surgery with residual visual impairment in his left eye but no residual facial droop and no further chest pain.

**Figure 1:**
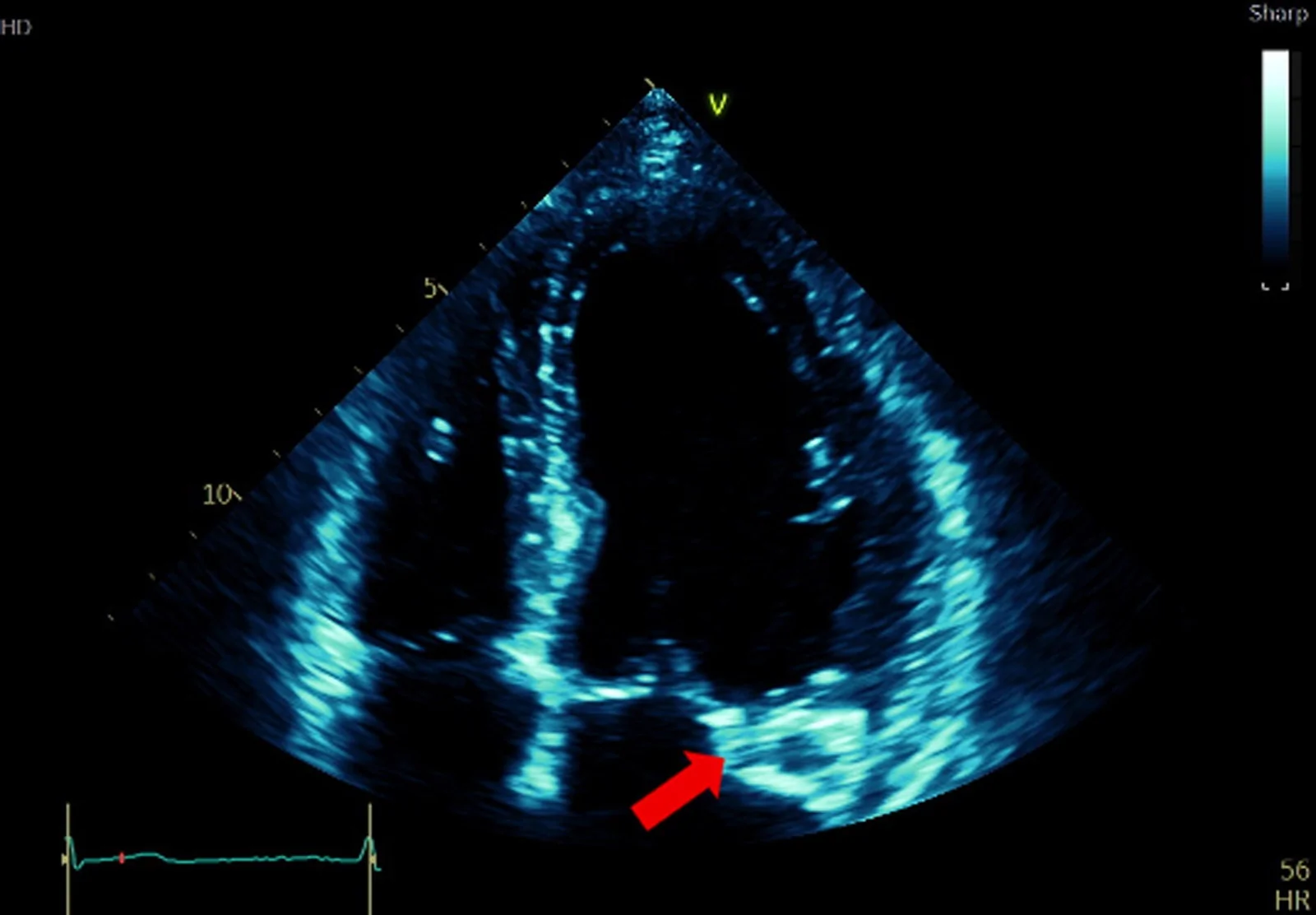
Transthoracic echocardiogram apical four-chamber view demonstrating a bright round echogenic structure with an echolucent core on the posterior mitral valve leaflet and annulus, thought to represent mitral annular calcification.

**Figure 2:**
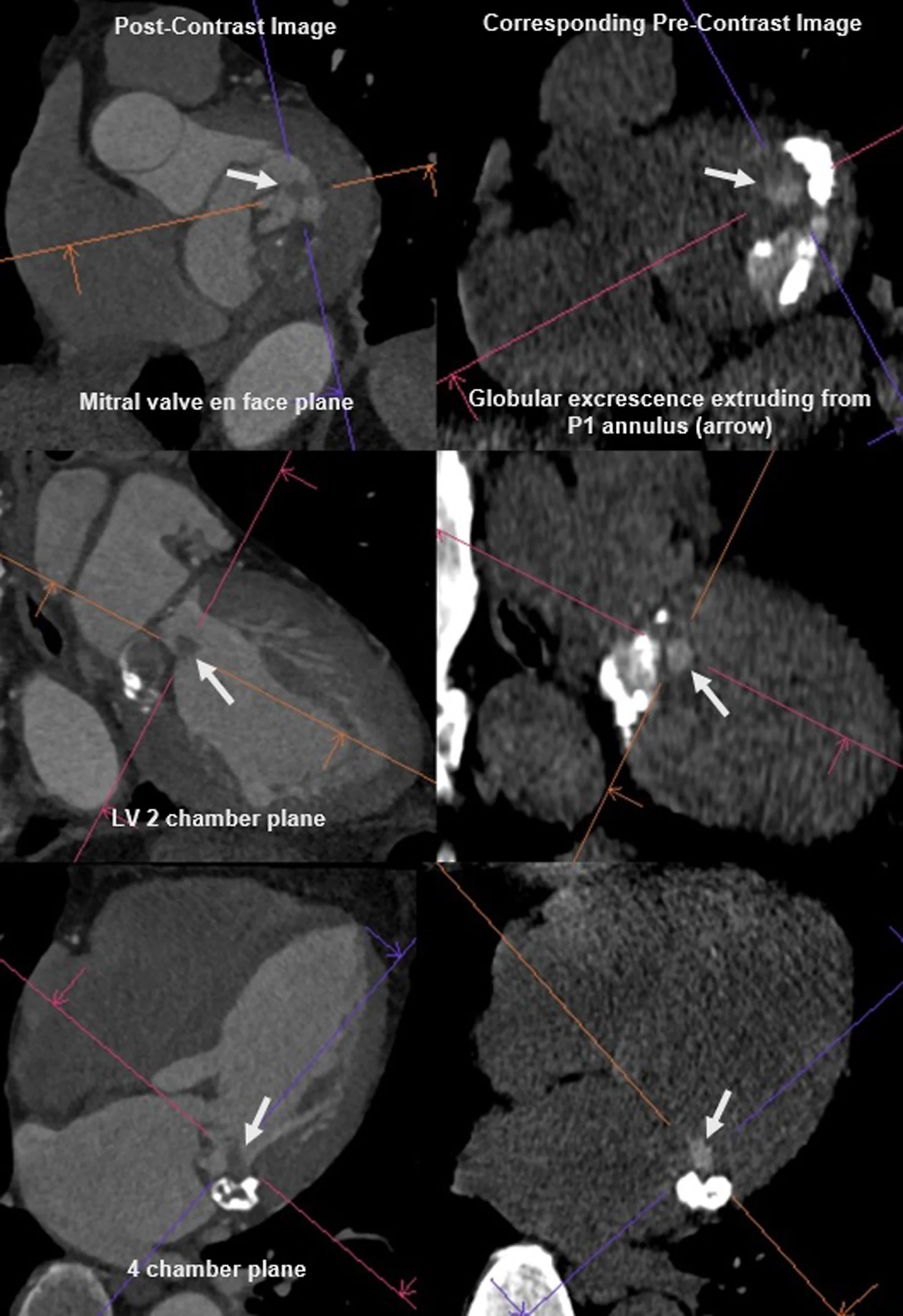
Cardiac computed tomography demonstrating severe caseous mitral annular calcification at the lateral annulus with a high-attenuation globular excrescence (arrows) extruding from the P1 annulus into the left ventricle.

**Figure 3:**
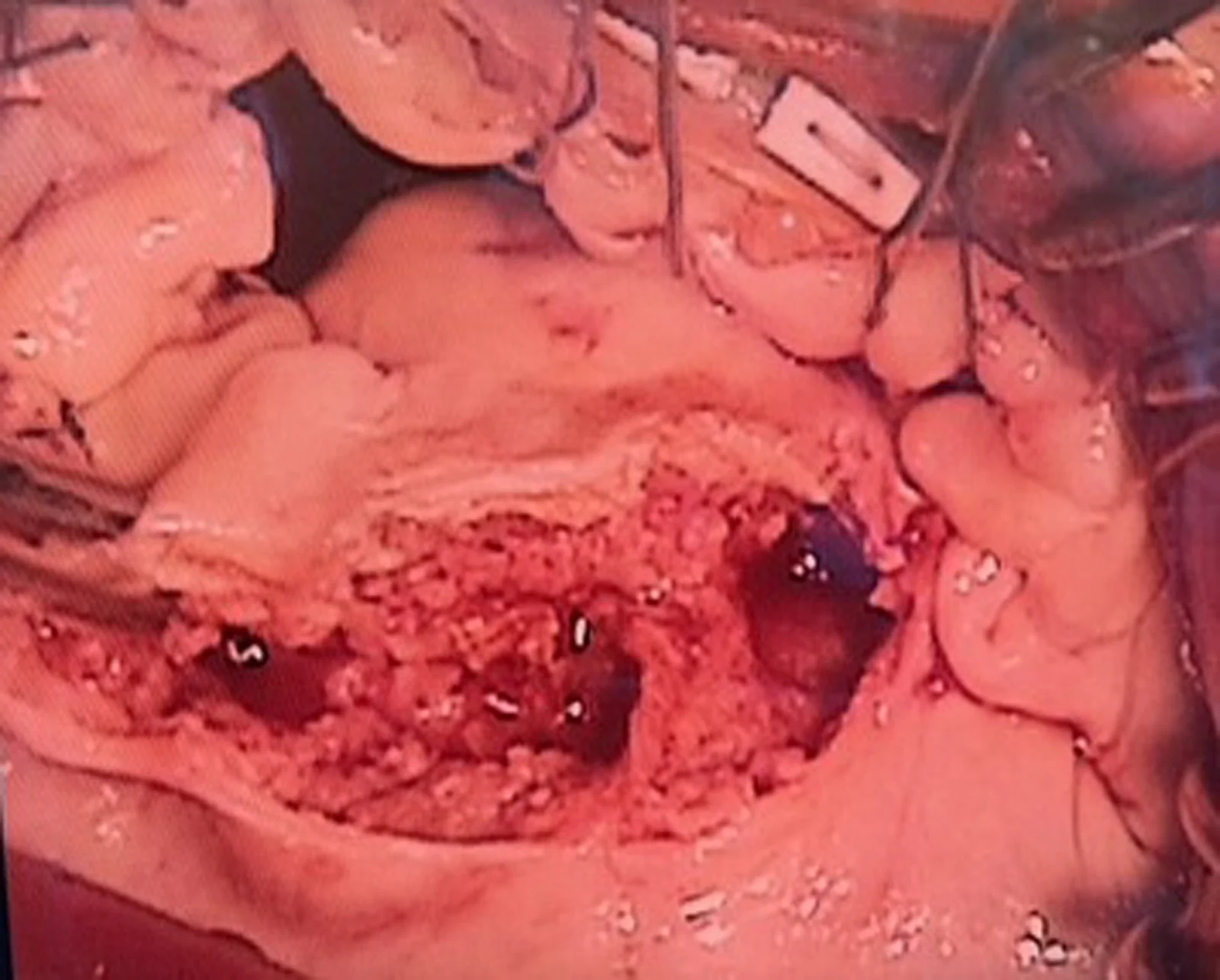
Operative findings revealed a rounded mass with dense peripheral calcification surrounding amorphous caseous material.

**Figure 4:**
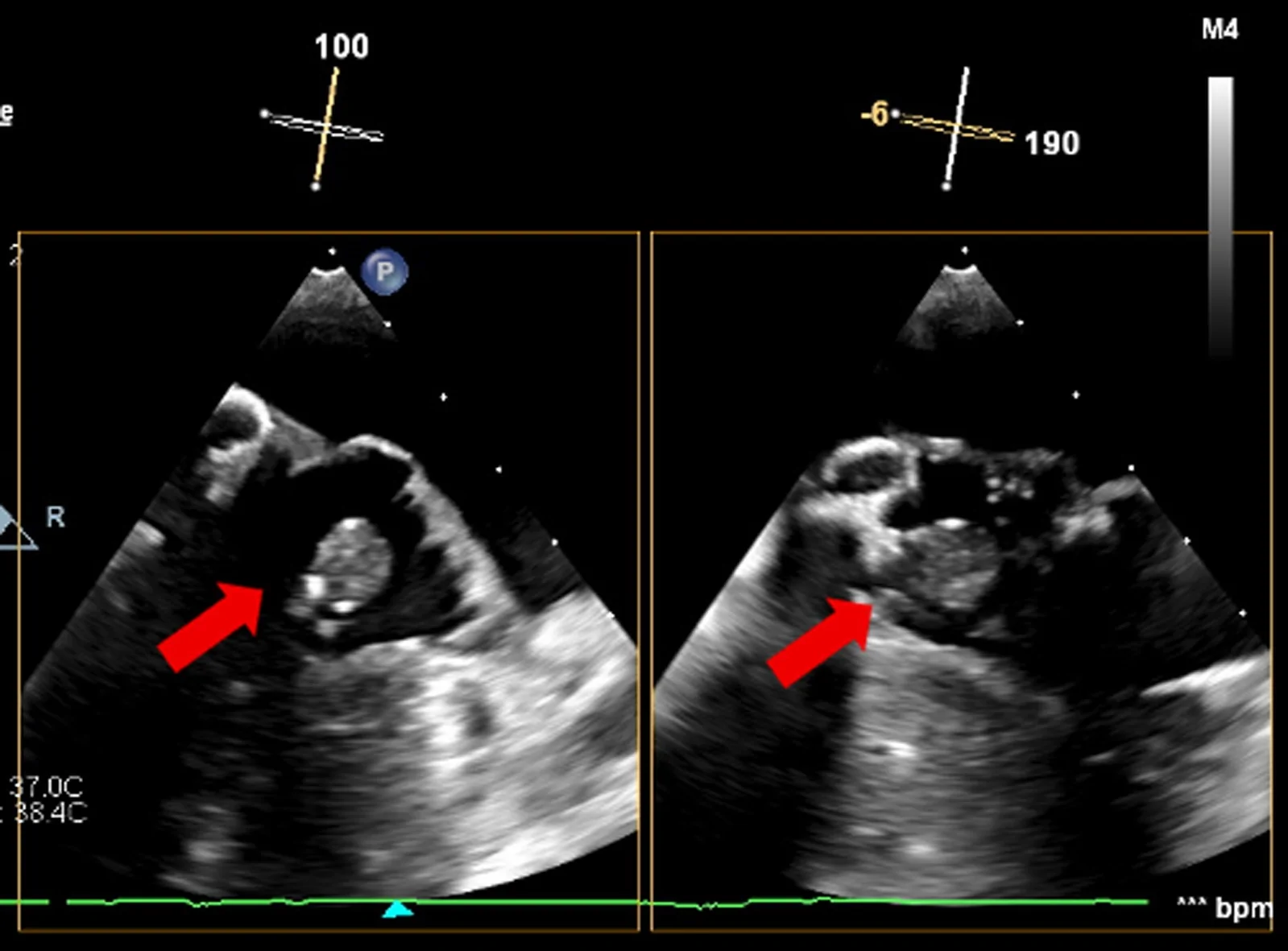
Transoesophageal echocardiogram demonstrated mitral annular calcification with a globular excrescence at the P1 annulus.

## Discussion

Mitral annular calcification is a chronic degenerative process. Risk factors include older age, female gender, hypertension, and renal failure.^1^ Caseous MAC is a rare variant of MAC with an estimated prevalence of 0.06%.^2^ Caseous MAC is characterized by a rounded mass with peripheral calcification and central echolucency, consistent with liquefaction necrosis. It comprises calcium, fatty acids, and cholesterol. Caseous MAC is usually found on the posterior mitral valve annulus, though in rare cases, it may affect the anterior annulus or both annuli. Differentials include abscesses, tumours, or infective vegetations.^3^ Caseous MAC is often diagnosed incidentally on cardiac imaging, as most patients remain asymptomatic. However, when sufficiently large, MAC may extend into adjacent tissues and result in mitral regurgitation, mitral stenosis, left ventricular outflow tract obstruction, or compression of the atrioventricular node leading to atrial arrhythmias or bradyarrhythmias. Thus, symptoms may include dyspnoea, palpitations, or syncope.^2^ Embolic complications, including stroke, acute coronary syndrome, and retinal artery occlusion, are rare. Postulated mechanisms of embolization include spontaneous fistulization with leakage of caseous material into the left atrium or ventricle, as in our case, embolization of necrotic debris from the caseous MAC core, and embolization of calcium and cholesterol particles.^4^ Several clinical features have been proposed as potential predictors of embolic events, including age > 70 years, female gender, hypertension, underlying hypertrophic and infiltrative cardiomyopathies, infective endocarditis, and larger or more mobile calcific deposits.^5,6^ However, these factors are based on observational data and should therefore be considered proposed predictors rather than validated risk factors. Cerebrovascular events have been reported in patients with caseous MAC, with one review reporting neurological events in up to 19% of patients.^4^ However, given that the available literature consists largely of case reports and small case series, the frequency of cerebrovascular embolization remains uncertain. Concurrent embolization to both the cerebral and coronary circulations appears to be exceptionally rare, with only one previous postmortem case identified in the literature.^7^

Transthoracic echocardiogram is the first-line modality for evaluating mitral valve anatomy and haemodynamics in MAC. Caseous MAC typically presents as a large, rounded echodense mass with a central echolucent core on the posterior annulus. However, acoustic shadowing due to calcification can limit assessment. Transoesophageal echocardiogram is often superior for assessing caseous MAC, due to its improved visualization of posterior cardiac structures.^8^ In our case, we were able to visualize leakage of caseous material into the left ventricle on TOE. Cardiac CT affords superior spatial resolution and capacity for tissue characterization. On CT, caseous MAC typically presents as an oval-shaped mass with peripheral calcification with high Hounsfield units and lack of contrast enhancement.^2^ Cardiac CT is particularly valuable for delineating the location and extent of calcification and distinguishing MAC from other lesions, such as thrombus and tumour. In this case, CT also identified the extruded caseous calcium as the likely source of embolism. Cardiac MRI can also aid in characterizing caseous MAC, which typically appears as a well-defined lesion with low-signal intensity on both T1- and T2-weighted sequences and no contrast enhancement on first-pass perfusion imaging.^9^

While conservative management is usually recommended for MAC, indications for surgery include mitral valve dysfunction or systemic embolization.

## Conclusion

We describe a rare case of caseous MAC complicated by concurrent stroke and MI. We highlight the importance of multimodal imaging in the diagnosis of caseous MAC and in identifying the underlying cause of the embolic events.

## Lead author biography


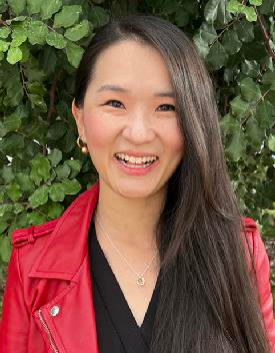
Jessica V. Yao is a cardiology imaging fellow and PhD candidate with a special interest in cardio-obstetrics and echocardiography.

## Data Availability

The data underlying this article are available in this article.
